# Adipose Lipolysis Regulates Cardiac Glucose Uptake and Function in Mice under Cold Stress

**DOI:** 10.3390/ijms222413361

**Published:** 2021-12-12

**Authors:** Youngshim Choi, Hyunsu Shin, Ziwei Tang, Yute Yeh, Yinyan Ma, Anil K. G. Kadegowda, Huan Wang, Long Jiang, Rakesh K. Arya, Ling Chen, Bingzhong Xue, Hang Shi, Oksana Gavrilova, Liqing Yu

**Affiliations:** 1 Division of Endocrinology, Diabetes, and Nutrition, Department of Medicine, University of Maryland School of Medicine, Baltimore, MD 21201, USA; Youngshim.choi@som.umaryland.edu (Y.C.); eyrevivi@gmail.com (Z.T.); Yu-Te.Yeh@som.umaryland.edu (Y.Y.); 13766808805@126.com (H.W.); skyiadx@hotmail.com (L.J.); rakeshumd19@gmail.com (R.K.A.); 2 Department of Animal and Avian Sciences, University of Maryland, College Park, MD 20742, USA; hyunsu.shin2012@gmail.com (H.S.); yinyan.ma@nih.gov (Y.M.); anilkadegowda@gmail.com (A.K.G.K.); 3 Center for Molecular and Translational Medicine, Institute for Biomedical Sciences, Georgia State University, Atlanta, GA 30303, USA; 4 Department of Physiology, University of Maryland School of Medicine, Baltimore, MD 21201, USA; Lichen@LurieChildrens.org; 5 Department of Biology, Center for Obesity Reversal, Georgia State University, Atlanta, GA 30303, USA; bxue@gsu.edu (B.X.); hshi3@gsu.edu (H.S.); 6 Mouse Metabolism Core Laboratory, the National Institute of Diabetes and Digestive and Kidney Diseases, National Institutes of Health, Bethesda, MD 20892, USA; oksanag@bdg10.niddk.nih.gov

**Keywords:** Abhd5, adipose lipolysis, cardiac energy substrates and remodeling, cardiac hypertrophy and dysfunction, cold adaptation

## Abstract

The heart primarily uses fatty acids as energy substrates. Adipose lipolysis is a major source of fatty acids, particularly under stress conditions. In this study, we showed that mice with selective inactivation of the lipolytic coactivator comparative gene identification-58 (CGI-58) in adipose tissue (FAT-KO mice), relative to their littermate controls, had lower circulating FA levels in the fed and fasted states due to impaired adipose lipolysis. They preferentially utilized carbohydrates as energy fuels and were more insulin sensitive and glucose tolerant. Under cold stress, FAT-KO versus control mice had >10-fold increases in glucose uptake in the hearts but no increases in other tissues examined. Plasma concentrations of atrial natriuretic peptide and cardiac mRNAs for atrial and brain-type natriuretic peptides, two sensitive markers of cardiac remodeling, were also elevated. After one week of cold exposure, FAT-KO mice showed reduced cardiac expression of several mitochondrial oxidative phosphorylation proteins. After one month of cold exposure, hearts of these animals showed depressed functions, reduced SERCA2 protein, and increased proteins for MHC-β, collagen I proteins, Glut1, Glut4 and phospho-AMPK. Thus, CGI-58-dependent adipose lipolysis critically regulates cardiac metabolism and function, especially during cold adaptation. The adipose-heart axis may be targeted for the management of cardiac dysfunction.

## 1. Introduction

Obesity and associated metabolic complications, including insulin resistance, type 2 diabetes, dyslipidemia, fatty liver disease, and cardiometabolic dysfunction, are a global health burden. Obesity is characterized by increased visceral fat, a type of adipose tissue that has the most detrimental metabolic effects. Adipose tissue can be classified into two major subtypes: white adipose tissue (WAT) and brown adipose tissue (BAT). WAT stores excess energy as triglycerides (TGs) in the unilocular cytosolic lipid droplets of white adipocytes. BAT dissipates metabolic energy as heat through the mitochondrial uncoupling protein 1 (Ucp-1) for nonshivering thermogenesis [1], though BAT also stores TGs in the multilocular small cytosolic lipid droplets of brown adipocytes. Another subtype of adipose tissue is called beige adipose tissue, which resides in the classical WAT depots but has features between WAT and BAT. Beige adipose tissue can be recruited to WAT depots by cold exposure and some agents, and this process is called white adipose browning [2,3]. Human adults possess substantial beige fat, though lacking grossly identifiable brown fat pads as seen in human infants and rodents [4,5,6]. Since BAT and beige fat convert metabolic energy into heat, agents and interventions recruiting BAT and beige fat may help combat obesity and associated metabolic disorders. 

The hydrolytic process that mobilizes cytosolic lipid droplet TG stores is called intracellular lipolysis [7]. During starvation or increased energy demands, intracellular lipolysis is stimulated, resulting in sequential cleavage of three fatty acyl chains from a TG molecule by three hydrolytic enzymes, adipose triglyceride lipase (ATGL), hormone-sensitive lipase, and monoglyceride lipase. ATGL is the rate-limiting enzyme and requires the coactivator CGI-58 to fully function [8,9]. The metabolic fate of fatty acids (FAs) released from lipid droplet lipolysis differs between WAT and BAT. FAs and glycerol from WAT lipolysis are released into the blood circulation to serve, respectively, as energy fuels for vital organs such as the heart and as the biosynthetic backbone for metabolic pathways such as hepatic gluconeogenesis. On the other hand, the lipolytic products in BAT are predominantly utilized by mitochondria within the same cells for heat generation. Despite having seemingly opposite functions, WAT and BAT coordinate in many aspects. For example, when BAT lipolysis is disrupted, animals rely on WAT lipolysis to maintain body temperature during fasting and cold stress [10,11,12].

Coordination exists not only among different adipose subtypes but also between adipose and non-adipose tissues. Recent research on adipose biology has discovered that adipose tissue can function as an endocrine organ via the secretion of adipokines, extracellular vesicles, special lipids, non-coding RNAs, and metabolites [13,14,15]. These versatile functions of adipose tissue place it at the crossroad of organ-organ communications in energy metabolism, normal physiology, and disease pathophysiology. In adults, normal cardiac function relies predominantly on the continuous supply of FAs as energy substrates [16]. Exogenous FAs delivered to cardiomyocytes in the heart are mainly derived from adipose lipolysis and/or hydrolysis of TGs in TG-rich lipoproteins by lipoprotein lipase (i.e., intravascular lipolysis) [7,17]. It has been shown that adipose-specific deletion of ATGL alters cardiac lipidome and prevents cardiac hypertrophy and heart failure in chow-fed mice under the transverse aortic constriction-induced pressure overload [18]. When these animals are subjected to chronic exercise, they show increased use of glucose in the heart as measured by ^18^F-fluorodeoxyglucose [19]. Adipose deletion of ATGL or its coactivator CGI-58 renders mice cold sensitive in the fasted state [10,12]. It remains completely unknown how the hearts of these animals respond to thermal stress such as cold exposure, a condition often used in thermoregulation and energy metabolism research and experienced by people in the winter and living in the northern hemisphere. Army soldiers are also often exposed to prolonged cold stress during training and on the battlefield. Cold stress has been linked to increasing cardiovascular events [20], but the underlying mechanisms remain elusive. The heart is an integral part of the thermoregulatory system since it disseminates heated blood to maintain body temperature. Additionally, the heart secretes atrial and brain-type natriuretic peptides (ANP and BNP) that have been shown to promote lipolysis and thermogenesis in the adipose tissue [21,22]. Cold stress is known to stimulate the sympathetic nervous system whose activation is the most powerful inducer of adipose lipolysis [1]. In this study, we demonstrated that adipose lipolysis is required for normal metabolic, structural, and functional adaptations of the heart to cold stress. The adipose-heart axis may be targeted to improve cardiac health, during cold adaptation, in particular.

## 2. Results

### 2.1. Selective Inactivation of CGI-58 in Adipose Tissue Reduces Blood Free Fatty Acids and Increases Whole-Body Glucose Utilization

**Figure 1:**
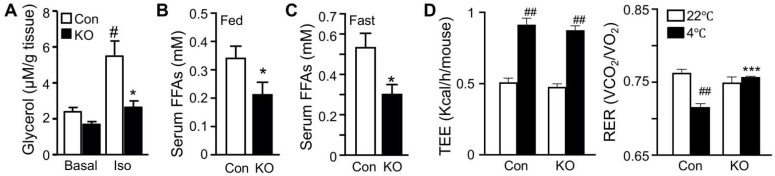

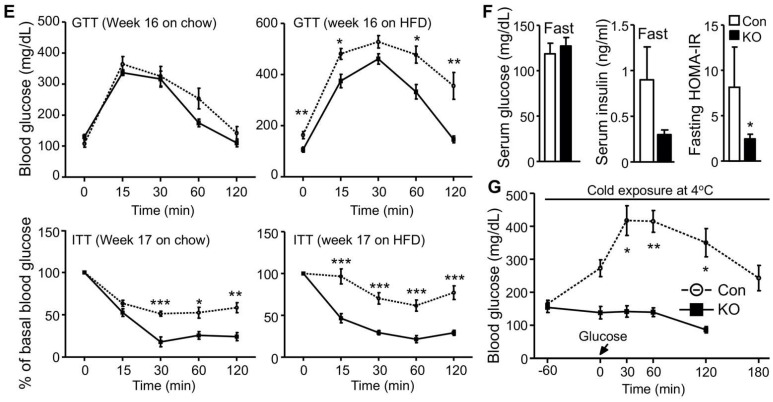
Deletion of adipose CGI-58 reduces serum FFA concentrations and increases whole-body glucose utilization. (**A**) Ex vivo lipolysis of iWAT explants from 14-week-old chow-fed mice (*n* = 5/group). Glycerol concentrations in the medium of cultured iWAT explants were measured after a 2 h incubation at 37 °C in the absence (Basal) or presence of isoproterenol (Iso). (**B**,**C**) Serum concentrations of FFAs in the 15-week-old HFD-fed mice in the fed state (**B**) and in the 17-week-old HFD-fed mice after overnight fasting (**C**) (*n* = 5–6/group). (**D**) The respiratory exchange ratio (RER) and the total energy expenditure (TEE) in 23-week-old FAT-KO and control mice fed the HFD ad libitum at room temperature and during cold exposure. The RER and TEE shown represent the data continuously collected for 3 h immediately before and 1 h after the switch of the housing temperature from 22 °C to 4 °C (*n* = 4–6/group). (**E**) Insulin and glucose tolerance tests in mice fed a chow diet (*n* = 5–6/group) or HFD starting at six weeks of age (*n* = 7–8/group) at room temperature. (**F**) Serum levels of insulin, glucose, and fasting homeostatic model assessment of insulin resistance (HOMA-IR) indexes in 17-week-old HFD-fed mice (*n* = 5–6/group). (**G**) Blood glucose levels of 11-week-old HFD-fed mice during acute cold exposure (*n* = 4–5/group). The mice were fasted for ~6 h during the daytime cycle and then exposed to 4 °C for 1 h prior to intraperitoneal injection of a bolus of glucose at 1.5 g/kg BW while remaining at 4 °C for the time indicated. * *p* < 0.05, ** *p* < 0.01, and *** *p* < 0.001 vs. genotype; # *p* < 0.05 vs. treatment; ## *p* < 0.001 vs. housing temperature by two-tailed unpaired Student’s *t*-test.

We have previously shown that FAT-KO relative to control mice gain similar weight on a regular chow or high-fat diet (HFD) and are completely resistant to the β-adrenergic receptor agonist isoproterenol-stimulated lipolysis as evidenced by the failed release of glycerol and FFAs into the blood circulation [10]. The WAT explant from FAT-KO mice was resistant to the isoproterenol-stimulated release of glycerol into the culture medium (Figure 1A). Importantly, chronic deficiency in adipose lipolysis lowered serum FFA levels under both fed and fasted states (Figure 1B,C). Considering the Randle (glucose-FA) cycle of metabolic fuel competition [23], we hypothesized that mice lacking adipose lipolysis are metabolically reprogrammed to utilize more glucose, especially under increased energy demanding conditions such as cold stress. To test this hypothesis, we placed the animals in a cold chamber. As expected, cold exposure increased total energy expenditure (TEE) in both genotypes (Figure 1D). The respiratory exchange ratio (RER) is an indicator of metabolic fuel sources. A higher value is indicative of carbohydrates versus fat as the preferred energy substrates. Consistently with our hypothesis, the RER was significantly higher in FAT-KO mice than the controls during cold exposure (Figure 1D). Increased use of carbohydrates is expected to improve glucose disposal. Indeed, FAT-KO mice compared to the controls showed better tolerance to glucose and were more sensitive to insulin-stimulated glucose disposal at normal housing temperature (Figure 1E). Despite no changes in serum glucose levels, serum insulin levels were consistently lower in FAT-KO mice than the control mice, resulting in a significant reduction in the homeostatic model assessment of insulin resistance index in FAT-KO mice (Figure 1F). Interestingly, a bolus of glucose injection cannot raise blood glucose levels in the FAT-KO mice during acute cold exposure (Figure 1G), suggesting an instant and complete tissue disposal of exogenous glucose by FAT-KO mice.

### 2.2. CGI-58 Deletion in the Adipose Tissue Increases Glucose Uptake in the Heart

**Figure 2:**
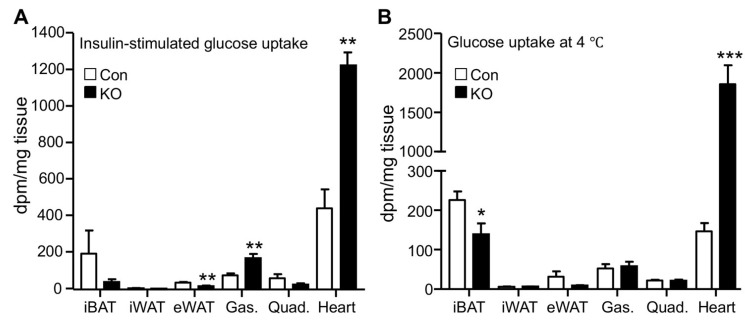
Adipose CGI-58 deficiency increases cardiac glucose uptake. (**A**) Tissue glucose uptake stimulated by insulin in 28-week-old HFD-fed mice housed at room temperature (*n* = 4–6/group). (**B**) Tissue glucose uptake in 20-week-old HFD-fed mice during acute cold exposure (*n* = 4–5/group). * *p* < 0.05, ** *p* < 0.01, and *** *p* < 0.001 vs. genotype by two-tailed unpaired Student’s *t*-test. iBAT, interscapular brown adipose tissue; iWAT, inguinal subcutaneous white adipose tissue; eWAT, epididymal white adipose tissue; Gas., gastrocnemius muscle; and Quad., quadriceps muscle.

Increased glucose disposal suggests enhanced tissue glucose uptake. Adipose and muscle tissues are major sites responsible for glucose disposal in peripheral organs. We, therefore, measured glucose uptake in these tissues by using the radioactive 2-deoxyglucose as a tracer. Under the insulin-stimulated condition, the hearts of FAT-KO mice relative to the control mice showed a >2-fold increase in glucose uptake (Figure 2A). Remarkably, cardiac glucose uptake increased >10-fold during acute cold exposure without insulin administration (Figure 2B). Relative to the heart, other tissues including interscapular brown adipose tissue, inguinal WAT, epididymal WAT, gastrocnemius muscle, and quadriceps muscle, showed lower glucose uptake per tissue weight under both insulin-stimulated and cold conditions, regardless of genotypes. Under the insulin-stimulated condition, gastrocnemius muscle and epididymal WAT had increased and decreased glucose uptake, respectively, in FAT-KO mice relative to control mice. Among the tissues examined, the heart was the only one showing increased glucose uptake per tissue wet weight in FAT-KO mice compared to the controls during cold stimulation.

### 2.3. Adipose CGI-58 Deficiency Increases Cardiac Expression of Natriuretic Peptides

**Figure 3:**
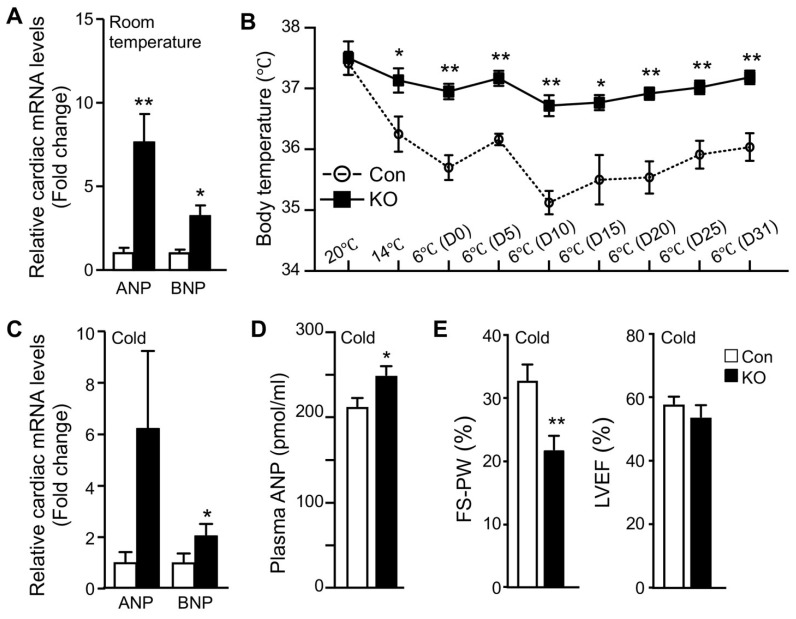
Adipose CGI-58 deficiency increases cardiac mRNA expression of natriuretic peptides and plasma ANP concentrations. (**A**) Relative mRNA levels of ANP and BNP in the hearts of 14-week-old HFD-fed mice housed at room temperature (*n* = 4–5/group). (**B**–**E**) Body temperature changes (**B**, *n* = 6–8), relative mRNA levels of natriuretic peptides (**C**, *n* = 5), plasma concentrations of ANP (**D**, *n* = 6–8), and echocardiography analysis (**E**, *n* = 6–8) of the HFD-fed mice that were subjected to cold acclimation (2 °C/day reduction in housing temperatures) for 7 days, followed by 32 days of cold exposure at 6 °C. * *p* < 0.05 and ** *p* < 0.01 vs. genotype by two-tailed unpaired Student’s *t*-test.

Normal adult hearts preferentially use FFAs as metabolic fuels. The increase in cardiac glucose uptake observed in FAT-KO mice prompted us to examine whether there was remodeling in the heart. Since cardiac remodeling is a chronic process, we measured atrial and brain natriuretic peptides (ANP and BNP), the two sensitive markers of cardiac remodeling and functional changes [24], in mice that were housed in normal room temperature. Indeed, there was a significant increase in cardiac mRNAs for both ANP and BNP (Figure 3A). Since cold stress-induced a >10-fold increase in cardiac glucose uptake (Figure 2B), we were interested in cold-associated cardiac remodeling. We subjected the mice to a long-term cold exposure regimen (7 days cold acclimation followed by 32 days of cold exposure at 6 °C) and then measured cardiac levels of mRNAs for ANP and BNP. The *ad libitum* fed FAT-KO mice at the age indicated can survive long-term cold exposure and maintain body temperature (Figure 3B). Interestingly, the body temperature was maintained at a higher level in the *ad libitum*-fed FAT-KO than control mice during chronic cold exposure. About one month after cold exposure, FAT-KO mice showed a substantial increase in cardiac levels of mRNAs for ANP and BNP (Figure 3C), and their plasma concentrations of ANP were also increased (Figure 3D). To determine whether these increases in ANP and BNP were associated with alterations in cardiac functions, we performed echocardiography. Interestingly, FAT-KO mice displayed a significant reduction in fractional shortening of LV posterior wall (FS-PW), though LV ejection fraction (LVEF) was not reduced (Figure 3E).

### 2.4. Adipose CGI-58 Deficiency Attenuates Cardiac Function during Cold Exposure

**Figure 4:**
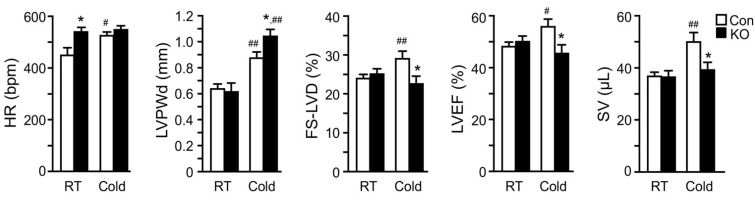
Deletion of adipose CGI-58 depresses cardiac responses to cold stress in chow-fed mice. Echocardiography analysis of cardiac structural and functional changes in 41–44-week-old chow-fed mice after cold acclimation (2 °C/day reduction in housing temperatures) for 7 days followed by housing at 6 °C for 26 days before analysis (*n* = 6–9/group). * *p* < 0.05 vs. genotype; # *p* < 0.05 and ## *p* < 0.01 vs. housing temperature by two-tailed unpaired Student’s *t*-test. HR, heart rate; LVPWd, left ventricular posterior wall thickness at end diastole; FS-LVD, left ventricular fractional shortening; LVEF, left ventricular ejection fraction; SV, stroke volume.

We speculated that high-fat feeding may have provided the heart with sufficient exogenous FFAs postprandially and slowed down the impact of adipose lipolysis deficiency on cardiac remodeling and function. In addition, a relatively long time is often needed for metabolic dysregulation to cause significant functional changes due to the generally large compensatory capacity of a heart. Given these, we measured cardiac functions in a cohort of middle-aged (10–11-months old) and chow-fed mice before and after 26 days of cold exposure (Figure 4). Interestingly, the heart rate was significantly higher in FAT-KO mice compared to the controls at the basal room temperature. Following the cold exposure, the heart rate was elevated in the control, but not KO mice. There was no significant difference in the heart rate between the two genotypes after cold exposure. The LV posterior wall thickness at end-diastole (LVPWd) increased in both genotypes following cold exposure, suggesting cold-induced LV hypertrophy. FAT-KO mice had an even greater response than control mice, causing a significant increase in LVPWd in these animals under cold stress. We did not observe any differences in the heart-to-body weight ratio following cold exposure (FAT-KO, 8.12% of BW vs. Control, 7.63% of BW), likely since the heart weight is generally not sensitive for LV hypertrophy in this model on a regular chow diet. There were no significant differences between the two genotypes in other echocardiographic parameters at the baseline (room temperature). Left ventricular fractional shortening (FS-LVD), LVEF, and stroke volume (SV) significantly increased in control mice following cold exposure, but these increases were not observed in FAT-KO mice. Following cold exposure, FS-LVD and LVEF were significantly lower in FAT-KO mice than controls. Therefore, it is evident that deletion of adipose CGI-58 disrupted the inotropic response of the heart to cold stress, which led to depressed LV systolic function. There were no significant changes in diastolic functions between the two genotypes or their responses to cold exposure (data not shown).

### 2.5. Adipose CGI-58 Deficiency Induces Pathological Remodeling of the Heart

**Figure 5:**
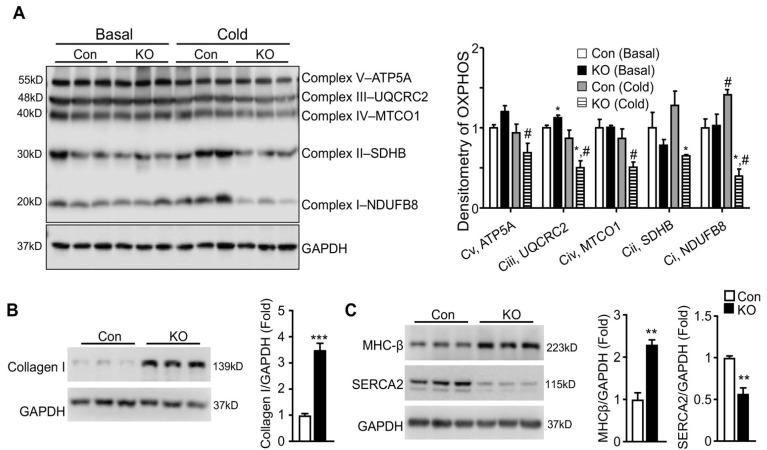
Deletion of adipose CGI-58 induces pathological remodeling of the heart. (**A**) Western blots and densitometry analysis of mitochondrial electron transport chain complex proteins in the hearts of 10–12-week-old chow-fed mice with or without cold exposure for seven days. (**B**,**C**) Western blots and densitometry analysis of biomarkers for pathological remodeling in the hearts of the mice described under Figure 4. * *p* < 0.05, ** *p* < 0.01, *** *p* < 0.001 vs. genotype; # *p* < 0.05 vs. housing temperature by two-tailed unpaired Student’s *t*-test. MTCO1, mitochondrially encoded cytochrome C oxidase 1; NDUFB8, NADH:ubiquinone oxidoreductase subunit B8; SDHB, succinate dehydrogenase complex iron sulfur subunit B (SDHB), UQCRC2, ubiquinol-cytochrome C reductase core protein 2.

To determine whether the depressed response of LV systolic functions to cold stress seen in FAT-KO mice was physiological or pathological, we first determined whether mitochondrial oxidative phosphorylation was affected in the FAT-KO mice since the heart as a major oxidative organ relies upon oxidative phosphorylation in mitochondria to generate energy and fulfill its vital function. We measured cardiac levels of representative proteins in five mitochondrial respiratory chain complexes at the early stage of cold exposure. Although no significant changes were observed for all of the representative proteins between the two genotypes at room temperature, levels of Complex I component NADH: ubiquinone oxidoreductase subunit B8 (NDUFB8), Complex II component succinate dehydrogenase complex iron sulfur subunit B (SDHB), and Complex III component ubiquinol-cytochrome C reductase core protein 2 (UQCRC2) were significantly reduced in FAT-KO mice under the cold stress (Figure 5A). Failed increases in cardiac mitochondrial respiratory chain proteins in the cold-exposed FAT-KO mice may not be able to meet the increased energy demand under chronic cold conditions, thereby causing cardiac remodeling. We, therefore, measured ventricular protein expression levels of genes related to pathological cardiac hypertrophy in FAT-KO mice after chronic cold exposure. There was a significant increase in collagen I and β-myosin heavy chain (MHC-β) and a significant decrease in sarcoplasmic reticulum Ca^2+^-ATPase (SERCA2) in FAT-KO mice compared to control mice (Figure 5B,C). This finding implies pathological remodeling of the heart in FAT-KO mice under cold stress.

### 2.6. Adipose CGI-58 Deficiency Increases Glucose Transporter Expression Levels and Cardiomyocytes in Hearts of Chow-Fed Mice after Chronic Cold Stress

**Figure 6:**
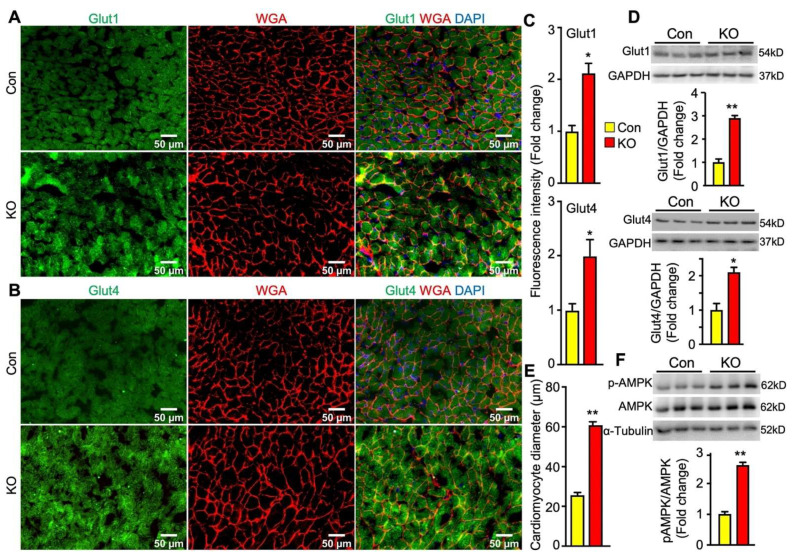
Adipose CGI-58 deficiency increases cardiomyocyte size and cardiac expression of proteins for Glut1, Glut4 and phosphorylated AMPK in chow-fed mice after cold stress. (**A**–**C**) Representative images and fluorescence intensity analysis of co-immunofluorescence staining of Glut1 or Glut 4 (green) with Alexa Fluor 594-conjugated WGA (red) in the cardiac tissues of the mice described under Figure 4. Quantification of fluorescence intensity was performed in three to five randomly selected microscopic fields per heart under 20x magnification (*n* = 4 mice/group). (**D**) Cardiac protein levels of Glut1 and Glut4 in the mice described under Figure 4. (**E**) The mean diameters of cardiomyocytes (µm) were measured from WGA-stained images in (**A**,**B**) with quantification of approximately 50–80 cells from 3 randomly selected image areas per heart under 20x magnification (*n* = 4 mice/group). (**F**) Levels of phosphorylated AMPK in the hearts of the mice described under Figure 4. * *p* < 0.05 and ** *p* < 0.001 vs. genotype by two-tailed unpaired Student’s *t*-test.

In the heart, glucose uptake is mainly controlled by glucose transporter 1 (Glut1) and Glut4. Glut1 is responsible for constitutive uptake of glucose [25], whereas Glut4 mediates insulin-stimulated glucose uptake. Both glucose transporters can translocate to the cell surface to mediate glucose uptake. To determine whether adipose lipolysis deficiency altered the abundance and subcellular localization of cardiac Glut1 and Glut4 in mice after cold exposure, we first performed immunofluorescence studies by using antibodies against Glut1 and Glut4 as well as Wheat Germ Agglutinin (WGA) that stains cells’ surfaces. It was observed that the intensity of fluorescence for both Glut1 and Glut4 proteins was much stronger and there was more colocalization of Glut1 or Glut4 with WGA in the hearts of FAT-KO mice than the controls after cold stress (Figure 6A–C). Consistent with increased fluorescence intensity, the heart of FAT-KO mice also expressed more Glut1 and Glut4 proteins when measured by immunoblotting (Figure 6D). When observing the WGA-stained sections under the microscope, we noticed apparent enlargement of cardiomyocytes and therefore performed morphometric analysis. Indeed, there was a significant increase in the diameters of cardiomyocytes in FAT-KO mice (Figure 6E), suggesting the development of cardiac hypertrophy in these animals after chronic cold exposure.

Energy fuel selection or switch has the potential to alter cellular energy status. AMP-activated protein kinase (AMPK) is the main energy sensor of cells [26]. In the cardiac tissue, activation of AMPK has been shown to promotes glucose uptake and glycolysis by recruiting Glut4 to the sarcolemmal membrane [27,28]. Interestingly, cardiac levels of phosphorylated AMPK were substantially increased in FAT-KO mice compared to the controls (Figure 6F). This finding suggests that AMPK activation may be a mechanistic link between adipose lipolysis inhibition and increased cardiac glucose utilization.

**Figure 7:**
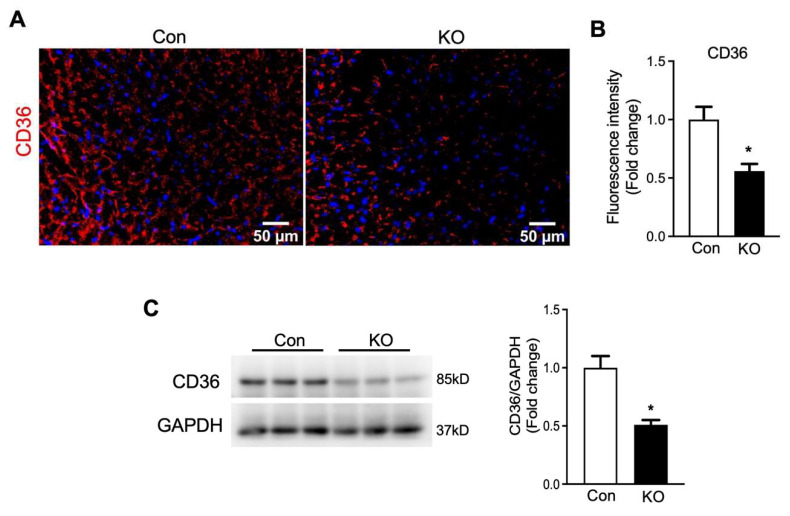
Deletion of adipose CGI-58 decreased cardiac CD36 protein level in chow-fed mice after cold stress. (**A**,**B**) Representative images and fluorescence intensity analysis of immunofluorescence staining of CD36 (red) with DAPI (blue) in the cardiac tissues of the mice described under Figure 4. Quantification of fluorescence intensity was performed in three to five randomly selected microscopic fields per heart under 20× magnification (*n* = 4 mice/group). (**C**) Cardiac protein levels of CD36 in the mice described under Figure 4. * *p* < 0.05 vs. genotype by two-tailed unpaired Student’s *t*-test.

In contrary to glucose transporters, the fatty acid transporter CD36 protein level was significantly decreased in the hearts of FAT-KO mice compared to control mice (Figure 7). The downregulation in cardiac CD36 protein, together with the decrease in circulating FFA levels and the increase in cardiac glucose uptake, indicates that the hearts of adipose lipolysis-deficient mice heavily rely on glucose catabolism for energy.

## 3. Discussion

Cold exposure has been widely used as a tool to study energy metabolism and thermoregulation. Since cold exposure stimulates heat production by brown/beige fats, it may mitigate overnutrition-induced metabolic disorders by increasing energy expenditure. On the other hand, cold is thermal stress that provokes instant and dramatic changes in the body. One of such changes is the activation of the sympathetic nervous system, which has at least two major consequences: (1) stimulation of adipose lipolysis for mobilization of energy stores to meet increased demands in heat generation and metabolism; and (2) stimulation of the cardiovascular system for distributing heat throughout the body. Since adult hearts prefer FAs as energy substrates, these two consequences downstream sympathetic activation appear to be coupled to coordinate whole-body thermogenesis. We, therefore, hypothesized that adipose lipolysis is essential for sustaining normal responses of the heart to cold stress. In the present study, we tested this hypothesis by using FAT-KO mice that are defective in adipose lipolysis. The major finding of this study was that hearts respond to adipose lipolysis deficiency by increasing glucose uptake. This metabolic fuel adaptation reduces the fractional shortening of the LV posterior wall, an early and sensitive parameter of systolic cardiac dysfunction, in adipose lipolysis-deficient mice fed an HFD. In the middle-aged mice (10–11 months) fed a regular chow diet, adipose lipolysis deficiency attenuated several inotropic responses of the heart to cold stress, resulting in depressed LV systolic function along with pathological cardiac remodeling and hypertrophy. Future studies are warranted to molecularly define how adipose lipolysis influences cardiac metabolism and function in a temporal manner and under various nutritional, stress, and disease conditions.

Early animal and human studies have shown that global inhibition of intracellular lipolysis using pharmacological agents such as acipimox increases glucose utilization in the normal and failing hearts [29,30,31,32]. Adipose lipolysis deficiency induced by genetic deletion of adipose ATGL also augments cardiac glucose uptake in mice subjected to exercise [19]. Our findings are consistent with this previous research, demonstrating a critical role of CGI-58-mediated adipose lipolysis in regulating cardiac glucose uptake. Additionally, our study was the first to show that glucose uptake increase is restricted to the heart among several peripheral tissues examined in adipose lipolysis-deficient mice under cold stress. There are a couple of plausible explanations for the heart-selective increase in glucose uptake under cold stress and lipolysis-deficient conditions. First, the heart is essential for survival in general and its energy supply must be prioritized. Glucose is relatively energy-efficient [29,33]. Second, the cardiovascular system is responsible for quickly disseminating the heat to maintain a body temperature that must be kept constant for normal metabolism. Given this, the heart is perhaps the most critical organ for whole-body thermogenesis [12,22]. An interesting question is how the heart turns on glucose uptake when adipose lipolysis is disrupted. We observed an increase in phosphorylation of the main cellular energy sensor AMPK in the hearts of FAT-KO mice, indicative of a status of energy deprivation. AMPK phosphorylation (i.e., activation) has been shown to increase myocardial glucose uptake by stimulating translocation of Glut4 and perhaps other glucose transporters to the sarcolemmal membrane [27,28,34]. Adipose lipolysis deficiency reduces blood FA levels and prevents stress-induced elevations in circulating FAs, which, together with reduced protein expression of the FA transporter CD36, may limit FAs available for cardiac mitochondria to oxidize, resulting in a fall of cellular ATP levels and concomitant AMPK activation.

During cold exposure, the heart can secrete increased amounts of natriuretic peptides to promote adipose lipolysis and thermogenesis [21,35]. A recent study suggests that this cardiac response is essential for mediating adipose lipolysis and non-shivering thermogenesis during cold adaptation [22]. Cold exposure activates the sympathetic nervous system and alters hemodynamics, which may be responsible for stimulating the cardiac secretion of natriuretic peptides. Here we found that inhibiting adipose lipolysis increases cardiac expression and secretion of natriuretic peptides during cold exposure. Although the underlying mechanism has yet to be determined, this finding highlights an interdependent relationship between the heart and adipose tissue during thermogenesis.

When adipose lipolysis is deficient, mice completely rely on dietary nutrients to survive the acute cold exposure [10,12]. In this study, we found that adipose lipolysis-deficient FAT-KO mice can survive chronic cold exposure after cold acclimation and when the food is continuously available, at least at the age tested (Figure 3B). During chronic cold exposure, these animals remain to completely rely upon food to survive the cold (unpublished observation). Interestingly, we consistently observed that FAT-KO mice relative to the controls display higher degrees of body temperature during chronic cold exposure. It is currently unknown whether this is related to the increased use of energy-efficient glucose or other thermogenic adaptations. A bolus of glucose injection cannot raise blood glucose levels in these animals under the cold condition (Figure 2G), demonstrating immediate and complete disposal of glucose. Many studies found that glucose can be an important substrate for cold-induced thermogenesis in BAT [36,37,38,39,40,41]. Here we observed that the heart relative to the other tissues examined (Figure 2B) has the highest glucose uptake in the cold exposed mice, which is consistent with a human study using the lipolysis inhibitor niacin [42]. These observations in animal and human studies imply a potential role of cardiac glucose utilization in defending body temperature against cold stress.

Normal adult hearts rely on the continuous supply of FAs as the main energy substrate [43]. It is well established that there is a critical metabolic and physiological link between the heart as a primary site of FA catabolism and the adipose tissue as the primary organ of FA storage. It is therefore critical to understand how changes in cardiac substrate availability and use trigger events that ultimately result in heart dysfunction. Under physiological conditions, mitochondrial oxidative phosphorylation of FAs is the main source of cardiac ATP [44,45]. Under pathological conditions such as LV hypertrophy, glucose utilization increases as a quick source of ATP since FA oxidation is reduced [46,47]. Lipolysis inhibitors have been used to investigate how reducing circulating FAs influences cardiac metabolism and function under a variety of pathological and physiological conditions, and the results have been controversial [29,45,48,49]. The discrepancy may result from differences in baseline metabolic and functional conditions of hearts as well as treatment durations. It should be pointed out that pharmacological inhibitors inhibit intracellular lipolysis in all cell types and their effects cannot be attributable solely to adipose lipolysis inhibition. Mice with ATGL deletion specifically in the adipose tissue have lipolysis deficiency restricted to this tissue type [12]. These animals display decreased systemic lipid oxidation and increased systemic glucose oxidation [19,50,51]. When subjected to exercise, adipose-specific ATGL knockout mice show attenuated cardiac hypertrophic response [19,52]. Notably, the substrate switch from FAs to glucose in the adipose ATGL knockout mice has a profound impact on cardiac and plasma lipidomes, which attenuates cardiac hypertrophy and heart failure in a transverse aortic constriction (TAC) pressure-overload model [18]. Another study suggests that the small-molecule ATGL inhibitor Atglistatin may have the therapeutical potential for the management of heart failure [53]. However, these findings from adipose ATGL knockout mice cannot be generalized for its coactivator CGI-58 since CGI-58 has an ATGL-independent function [54]. The role of adipose CGI-58 has never been linked to cardiac metabolism and function under any conditions. Additionally, there were no studies that have evaluated the role of adipose ATGL or CGI-58 in regulating cardiac responses to cold stress. Others have shown that increased glucose utilization can cause LV hypertrophy and cardiac dysfunction as summarized in an elegant review [33]. LV hypertrophy may in turn exacerbate glucose utilization. High glucose was shown to induce mitochondrial dysfunction in isolated cardiomyocytes, diminishing cardiomyocyte respiration [55]. Over a long period, adaptive glycolytic metabolism cannot keep providing sufficient amounts of energy for the heart and may ultimately lead to cardiac dysfunction [33]. Our data from FAT-KO mice largely agree with this scenario. These animals relative to their controls already expressed reduced levels of several mitochondrial OXPHOS proteins in the hearts at young (~three months of) age (Figure 5A) and showed many metabolic abnormalities before six months of age. At their middle age (10–11 months old), FAT-KO mice displayed increased LV posterior wall thickness at end-diastole, cardiac hypertrophy, and depressed cardiac functions. Our observations suggest that metabolic abnormalities precede functional changes in the hearts of adipose lipolysis-deficient FAT-KO mice. 

The cardiac hypertrophy seen in FAT-KO mice appears to be pathological since it was associated with structural remodeling of the ventricular wall as evidenced by increased MHC-β and reduced SERCA2 proteins in the heart. These changes in MHC-β and SERCA2 are expected to adversely affect cardiac contractility and Ca^2+^ cycling [56] and are associated with failing hearts [57,58,59,60]. In addition, cardiac hypertrophy in FAT-KO mice was associated with increased expression of type 1 collagen, indicative of cell death and loss of cardiomyocytes, which is expected to impair contraction and relaxation of ventricles [61]. Thus, CGI-58-mediated adipose lipolysis is required for normal cardiac adaptations to the chronic cold stress. Our findings imply that adipose lipolysis could be targeted acutely or chronically to switch cardiac metabolic substrates, an important factor known to shape cardiac remodeling in disease causes- and time-dependent manners [62,63]. Lipolysis inhibitors such as niacin and acipimox are currently used clinically. As mentioned above, these inhibitors inhibit intracellular lipolysis in all cell types and their effects on cardiac remodeling and functions remain controversial likely due to their limited cell-type specificity and the differences in experimental and trial conditions and durations. Adipose-specific lipolysis inhibitors are not currently available for clinical use. The development of such lipolysis inhibitors may have the potential to change the course of pathological ventricular remodeling by regulating the availability of metabolic substrates of hearts in a disease cause- or stage-specific manner. In addition, such adipose-specific lipolysis inhibitors would not affect other tissues locally, avoiding potential lipotoxicity associated with lipolysis inhibition in non-adipose tissues.

Reginal and systemic hypothermia procedures have been applied before and after ischemia in animals and humans with ST elevation myocardial infarction (STEMI), hoping to reduce infarct size and reoxygenation injury [64,65,66,67,68,69,70]. While regional and intracoronary hypothermia has been consistently shown to reduce infarct size and reoxygenation/reperfusion-associated tissue injury and functions, systemic applications of therapeutic hypothermia seem to have limited benefits likely due to slow reduction in the local temperature of the ischemic and re-perfused cardiac tissue as well as the adverse effects of systemic hypothermia on hemostasis and adrenergic activation. Lowering the local temperature of ischemic and re-perfused tissues slows down tissue damage likely by reducing metabolic rates, calcium overload and oxidative stress, among others [66,71]. Interestingly, it has been reported that glucose may act as a free radical scavenger and as a metabolic substrate for glycolytic ATP production to protect ischemic heart tissue from subsequent reoxygenation damage and arrhythmias [71,72]. Fatty acids seem to have opposite effects in this regard due to the opposite roles of glucose and fatty acids in regulation of cellular cyclic AMP [71]. Systemic cold exposure is well known to activate the sympathetic nervous system, thereby activating adipose lipolysis. As a result, more free fatty acids are released to the bloodstream from adipose stores, which may harm ischemic hearts acutely via their lipotoxicity. In our study, inhibiting adipose lipolysis promotes glucose utilization in the heart and makes the animals more sensitive to cold exposure. Perhaps, short-term applications of a lipolysis inhibitor such as niacin or acipimox in combination with therapeutic hypothermia may rapidly reduce body temperature and boost glucose utilization during ischemic attack and reperfusion in patients with STEMI.

## 4. Materials and Methods

### 4.1. Animals and Diets

FAT-KO mice were generated by crossing CGI58-floxed mice with mice expressing Cre recombinase under the control of Adipoq promoter [B6;FVB-Tg(Adipoq-cre)1Evdr/J mice, The Jackson Laboratory, Stock #: 010803]. These mice had no CGI-58 protein expression in whole adipose tissue [10]. All animal experiments were performed using male FAT-KO mice and their littermate controls (i.e., homozygous CGI-58-floxed mice without Adipoq-cre transgene). Mice were fed a regular chow diet (Diet 5010, LabDiet, St. Louis, MO, USA) or a high-fat diet (HFD) (D12492, Research Diets, New Brunswick, NJ, USA) throughout the study beginning at six weeks of age. The mice used in this study were ~three to six-month-old, which represent the mature adults of this species according to Jackson Laboratory classification unless otherwise specified. The specific age of the mice used for each experiment is indicated in the figure legend. All mice were housed in a pathogen-free barrier facility with a 12 h light/dark cycle (light from 06:00 to 18:00) and had ad libitum access to water and food in rooms at 22 °C or in an environmental rodent chamber during cold exposure. At the termination of the study, mice were fasted for 4 h during the light cycle and then deeply anesthetized with isoflurane (5%) via inhalation. After the deep anesthesia, the animals were euthanized by complete blood drawing and tissue exsanguination. The tissues were rapidly excised, weighed, and either snap-frozen in liquid nitrogen before being stored at −80 °C or processed for histology. All experimental procedures were approved by the Institutional Animal Care and Use Committees at the University of Maryland College Park, Georgia State University, the National Institute of Health, and the University of Maryland Baltimore.

### 4.2. Indirect Calorimetry

The metabolic phenotyping was performed using the Comprehensive Lab Animal Monitoring System (CLAMS) as we have described previously [10].

### 4.3. Glucose and Insulin Tolerance Tests

For glucose tolerance test (GTT), mice were fasted overnight (16 h) and subsequently injected (i.p.) with a bolus of a glucose solution at a dose of 1.5 g/kg body weight (BW). Blood glucose levels were measured after a tail cut at 0, 15-, 30-, 60-, and 120-min post glucose injection by using a Bayer Contour Glucometer (Bayer Healthcare, LLC.). For the insulin tolerance test (ITT), mice were intraperitoneally injected with the recombinant human insulin (Millipore) diluted in saline after a 6h fast during the light cycle at a dose of 0.75 U/kg BW. Blood glucose levels were measured as described for GTT.

### 4.4. Blood Analysis

Plasma concentrations of ANP were measured using Mouse Atrial Natriuretic Peptide ELISA kit (Cusabio, Houston, TX, USA) following the manufacturer’s instructions. Serum concentrations of nonesterified free fatty acids (NEFAs) and glycerol were measured using a NEFA-HR (2) kit (Wako Diagnostics, Mountain View, CA, USA) and Free Glycerol Reagent (Sigma, St. Louis, MO, USA). Blood concentrations of glucose and insulin were determined using Bayer Contour Glucometer (Bayer Healthcare, LLC., Whippany, NJ, USA) and Insulin ELISA Kit (Millipore, Burlington, MA, USA).

### 4.5. Tissue Glucose Uptake Assay

For insulin-stimulated glucose uptake, mice fasted overnight for 16h and then injected intraperitoneally with human insulin at 0.75 mU/kg BW and 10 μCi of [^14^C]-2-deoxyglucose (PerkinElmer, Akron, Ohio, USA). Forty-five minutes following injection, the mice were anesthetized with isoflurane (5%) and tissues were collected. To assess glucose uptake under the cold stress, mice were fasted overnight for 16h and then subjected to 4 °C for 30 min before the injection of a 10 μCi of [^3^H]-2-deoxyglucose (PerkinElmer) as a tracer. The mice remained at 4 °C for an additional 45 min, then were anesthetized with isoflurane (5%) and tissues collected. Typically, deoxyglucose is transported into tissues and phosphorylated to 2-deoxyglucose 6-phosphate. Tissue contents of deoxyglucose phosphate were used to reflect the tissue glucose uptake efficiency. In order to determine tissue deoxyglucose phosphate content, tissues were homogenized and deoxyglucose phosphate was separated from deoxyglucose using an ion-exchange column (Chromatography Columns #731-6211, Bio Rad) and then counted as described [73].

### 4.6. Gene Expression Analysis

Total RNA was isolated from the heart tissue using TRIzol (Invitrogen, Waltham, MA, USA) according to the manufacturer’s protocol and treated with DNase I (Invitrogen) to remove genomic DNA. The cDNA was synthesized using TaqMan Reverse Transcription Reagents (Applied Biosystems, Waltham, MA, USA). The mRNA expression levels were measured by quantitative real-time PCR analysis using SYBR Green Real-time PCR Master Mix (Invitrogen). Reactions were carried out in triplicate for each biological sample. Changes in mRNA abundance were determined using the relative Ct method. Glyceraldehyde 3-phosphate dehydrogenase (GAPDH) was used as an internal invariant control.

### 4.7. Echocardiography

Echocardiography of mice was performed as previously described [74]. Briefly, animals were anesthetized by inhalation of 1.2 to 1.5% isoflurane in pure oxygen, with the core temperature maintained at 37.5 °C using a thermoregulated platform (THM 150; Indus Instruments, Webster, TX, USA). Transthoracic 2D-guided M-mode images of the left ventricle (LV) and the ascending aorta, as well as pulse-wave Doppler images of the ascending aortic flow and mitral valve inflow, were obtained by using a high-resolution ultrasound biomicroscope (Vevo 2100; VisualSonics, New York, NY, USA) equipped with a 30-MHz scan head. Data were calculated according to the generally accepted formulas as described previously [75].

### 4.8. Immunoblotting

Frozen heart tissues were homogenized in a radioimmunoprecipitation assay (RIPA) buffer supplemented with a protease inhibitor cocktail and a phosphatase inhibitor cocktail (Sigma). Protein concentrations of tissue homogenates were measured using a bicinchoninic acid assay (Pierce, Waltham, MA, USA). Samples were separated on sodium dodecyl sulfate-polyacrylamide gel electrophoresis (SDS-PAGE) and transferred by semi-dry blotting onto a nitrocellulose membrane (Bio-Rad, Hercules, CA, USA). Blots were blocked in 5% nonfat milk in Tris-buffered saline with Tween 20 and incubated overnight at 4 °C with antibodies against Glut1 (21829-1-AP, Proteintech Technology, Rosemont, IL, USA), Glut4 (MA5-17176, Thermo Scientific, Waltham, MA, USA), OXPHOS proteins (ab110413, Abcam, Boston, MA, USA), MHC-β (ab50967, Abcam), SERCA2 (ab150435, Abcam), Collagen I (ab21286, Abcam), phospho-AMPKα (Thr172) (CST #2535, Cell Signaling Technology, Boston, MA, USA), AMPKα (CST #2532, Cell Signaling Technology), CD36 (ab64014, Abcam), α-Tubulin (CST #2144, Cell Signaling Technology) and GAPDH (CST #2218, Cell Signaling Technology). After washing, blots were exposed to secondary antibodies conjugated to horseradish peroxidase for 1 h and developed in enhanced chemiluminescence (ThermoFisher Scientific, Waltham, MA, USA). The protein signals were detected with a Bio-Rad or SYNGENE ChemiDoc System and captured images were analyzed with ImageJ software (NIH, Bethesda, MD, USA).

### 4.9. Immunofluorescent Staining

Immunofluorescence staining was performed on frozen or paraffin-embedded tissue sections. Tissue sections were stained with 5 µg/mL of Wheat Germ Agglutinin (WGA), Alexa Fluor^®^ 594 Conjugate (W11262, Thermo Fisher Scientific, Waltham, MA, USA) for 10 min at room temperature and then with Glut1, Glut4, and CD36 antibodies in PBS containing 5% BSA at 4 °C overnight. The next day, the sections were incubated at room temperature for 1 h with donkey anti-rabbit Alexa Fluor 488 or donkey anti-mouse Alexa Fluor 488 secondary antibody. Cell nuclei were then stained with 4′,6-diamidino-2-phenylindole (DAPI) for 15 min at room temperature. At least three images per specimen were obtained on an Olympus IX50 (Olympus, Center Valley, PA, USA) fluorescent microscope. Cardiomyocyte diameters in each heart were measured in ~50–80 cells from three randomly selected WGA-stained areas.

### 4.10. Statistical Analysis

Statistical analysis was performed using Prism 8 (GraphPad, San Diego, CA, USA). Data were expressed as MEAN ± SEM. Statistical significance was tested using the two-tailed unpaired Student’s t-test between the two groups to be compared. The difference between the groups was considered statistically significant if the *p-* value was less than 0.05.

## 5. Conclusions

In conclusion, adipose lipolysis deficiency induced by genetic deletion of adipose CGI-58 in mice increases cardiac glucose uptake, in particular during cold exposure. This metabolic adaptation is accompanied by pathological cardiac remodeling and hypertrophy, attenuating functional responses of the heart to chronic cold stress. Moreover, adipose lipolysis deficiency augments cardiac natriuretic peptide expression. Heart-derived natriuretic peptides are well-established activators of adipose lipolysis. Therefore, there exists a mutual regulation between cardiac natriuretic peptides and adipose lipolysis. Although the pathophysiological significance of this mutual regulation is not fully understood, existing data including those from this study indicate that normal crosstalk between adipose tissue and heart is crucial for maintaining metabolic and functional health of both tissues.

## Data Availability

The data presented in this study are available on request from the corresponding author.
